# Item response modeling: a psychometric assessment of the children’s fruit, vegetable, water, and physical activity self-efficacy scales among Chinese children

**DOI:** 10.1186/s12966-017-0584-x

**Published:** 2017-09-16

**Authors:** Jing-Jing Wang, Tzu-An Chen, Tom Baranowski, Patrick W.C. Lau

**Affiliations:** 1 Mass Sports Research Center, China Institute of Sport Science, Beijing, China; 2 HEALTH Research Institute, University of Houston, Houston, TX 77204 USA; 3 Children’s Nutrition Research Center, Department of Pediatrics, Baylor College of Medicine, Houston, TX 77030 USA; 4 Department of Physical Education, Faculty of Social Sciences, Hong Kong Baptist University, Academic and Administration Building, 15, Road, Kowloon Tong, Hong Kong, China

**Keywords:** Self-efficacy, Eating behaviors, Physical activity, Item response modeling, Differential item functioning

## Abstract

**Background:**

This study aimed to evaluate the psychometric properties of four self-efficacy scales (i.e., self-efficacy for fruit (FSE), vegetable (VSE), and water (WSE) intakes, and physical activity (PASE)) and to investigate their differences in item functioning across sex, age, and body weight status groups using item response modeling (IRM) and differential item functioning (DIF).

**Methods:**

Four self-efficacy scales were administrated to 763 Hong Kong Chinese children (55.2% boys) aged 8-13 years. Classical test theory (CTT) was used to examine the reliability and factorial validity of scales. IRM was conducted and DIF analyses were performed to assess the characteristics of item parameter estimates on the basis of children’s sex, age and body weight status.

**Results:**

All self-efficacy scales demonstrated adequate to excellent internal consistency reliability (Cronbach’s α: 0.79-0.91). One FSE misfit item and one PASE misfit item were detected. Small DIF were found for all the scale items across children’s age groups. Items with medium to large DIF were detected in different sex and body weight status groups, which will require modification. A Wright map revealed that items covered the range of the distribution of participants’ self-efficacy for each scale except VSE.

**Conclusions:**

Several self-efficacy scales’ items functioned differently by children’s sex and body weight status. Additional research is required to modify the four self-efficacy scales to minimize these moderating influences for application.

## Background

The alarming rates of chronic diseases have been attributed to dietary habits and physical activity (PA) patterns [1, 2]. Increasing fruit and vegetable consumption, replacing sweetened beverages with water, and engaging in sufficient PA facilitate chronic disease prevention [3]. Furthermore, the dietary and PA practices tend to initiate and develop during childhood at which time it is desired to foster healthier habits [4].

Self-efficacy, a central component of Bandura’s social cognitive theory, is concerned with people’s beliefs and capabilities to perform or maintain actions at designated levels and has been advanced as an important individual determinant of human behavior [5]. Perceived self-efficacy for fruit, vegetable, and water intakes and PA were strong predictors of corresponding behaviors [6, 7] and key variables mediating change from interventions [8, 9]. Increasing self-efficacy has been adopted as an effective intervention strategy [10–12]. Questionnaires on self-efficacy for fruit (FSE), vegetable (VSE), and water intakes (WSE), and PA (PASE) in existing studies have varied in numbers and types of items, subscales and psychometric characteristics. For example, PASE was measured by a 8-item PASE [13–15] developed by Motl and colleagues [16] or a modified version [17], while other studies [18, 19] used the scale developed by Saunders et al. [20], or self-constructed questionnaires [12, 21]. While, some of these self-efficacy scales showed acceptable/adequate internal consistency (Cronbach’s alpha coefficient (α) higher than 0.70) and test-retest reliability (TRT larger than 0.60) [13–20], others did not [22].

Valid and reliable measures are needed to test the associations between self-efficacy and behavior and to examine the possible mediating effect of self-efficacy in behavior change programs. Levels of self-efficacy have been reported to be significantly different by children’s sex, age, and body weight status [23–25]. True differences in the validity of the measurement scale may make it difficult to compare parameter estimates across these different groups when comparing the results across studies. Furthermore, understanding the group-related differences in item validity across demographic or body weight status groups could help design interventions tailored to specific items in different groups and thereby enhance program effectiveness.

Classical test theory (CTT), the traditional method for evaluating scales, is sample-dependent, and thereby cannot assess the functioning of item responses across different groups. Item response modeling (IRM) is a psychometric analysis method that provides model-based measurements. IRM links the individuals’ difficulty of response to each item, provides the distribution of respondents across the scale, and enables differential item functioning (DIF) analysis [26]. While, item functioning of children’s FSE and VSE has been evaluated by sex and ethnic groups in American children [27], no one has analyzed item functioning across age and body weight status groups for FSE and VSE, nor conducted this kind of analysis for WSE and PASE, nor among Chinese children.

This study evaluated the psychometric properties of FSE, VSE, WSE, and PASE and investigated item differences in their psychometric properties across sex, age, and body weight status groups using IRM and DIF.

## Methods

### Participants

The sample was from the validation study of the Physical Activity Questionnaire for Older Children among Chinese children [28]. Children (*n* = 798, 55.8% males) aged 8-13 years old were recruited from six Hong Kong primary schools that agreed to participate in the study. The schools were located in different administrative districts with varied socio-economic status (SES) (two from high SES, one from medium SES, and three from low SES districts) according to local statistics [29]. Students were excluded if they had any contraindication to participating in PA or eating a normal diet. A subsample of 94 children (54.3% males) was randomly selected to complete the questionnaires twice within 7-10 days to assess the scale test-retest reliability. The ethic committee of Hong Kong Baptist University approved this study.

### Measures

A standard translation and back translation procedure was used with three bilingual language speakers (i.e., English and Cantonese). Minor wording revisions were made according to cognitive interviewing feedback from five primary students to ensure that target children could understand the instructions and items. All participants completed the questionnaire set in schools under the administration of research assistants.

#### Body weight status

Children’s height and weight, measured by physical education teachers, were retrieved from the latest school records. Height was measured to the nearest 0.1 cm and weight was measured to the nearest 0.1 kg. Body mass index (BMI, kg/m^2^) was calculated as weight in kilograms divided by height in meters squared. According to international age- and sex- specific cutoff points, body weight status of participating children were classified into underweight [30], healthy, overweight and obese [31] groups based on their BMI values.

#### Self-efficacy for fruit (FSE), vegetable (VSE) and water (WSE)

Validated self-efficacy scales for fruit, vegetable and water intakes were used to assess children’s FSE, VSE and WSE [32]. The scales consisted of 12, 8, and 5 items with dichotomous “sure” and “not sure” response categories and demonstrated acceptable internal consistency for FSE (α = 0.75) and VSE (α = 0.70) and marginal level of internal consistency for WSE (α = 0.55) in an American sample [32]. Construct validity was assessed through correlation among the self-efficacy scores and fruit and vegetable consumption, preferences and outcome expectancies (*r* = 0.10-0.21) [32]. Each item of the self-efficacy scales asked about the participant’s confidence in consuming fruit, vegetables or water under diverse circumstances. A FSE sample item included “How sure are you that you can eat 1 portion of fruit for a snack at home at least four days a week?” A VSE sample item included: “How sure are you that you can eat 3 portions of vegetables at least 4 days a week?” A WSE sample item included “How sure are you that you can drink 4 glasses or bottles of water for at least one day?” Considering item response difficulty, all items featured three response options in this study (1 = I am not sure; 2 = I am a little bit sure; 3 = I am very sure). The internal consistency in this sample was 0.86, 0.85, 0.79 for FSE, VSE, and WSE, respectively.

#### Self-efficacy for physical activity (PASE)

Children’s PASE was assessed by a validated Physical Activity Self-efficacy scale [33]. The scale had 12 items and demonstrated adequate internal consistency (α = 0.81) in the original validation study [33]. Weak but comparable correlations (*r* = 0.09-0.11) were found between PASE and minutes of moderate- to vigorous- activity. Similar to the FSE, VSE and WSE, children responded how sure they were that they could engage in PA in various conditions with a 3-response category (1 = I am not sure; 2 = I am sure a little; 3 = I am sure a lot). Sample items included “How sure are you that you can be physically active more than 30 minutes for at least 4 days a week, even when the weather outside is bad?” “How sure are you that you can ask your friends to be physically active with you more than 30 minutes for at least 4 days a week?” The scale in this sample presented excellent internal consistency (α = 0.91).

### Statistical analyses

#### Classical test theory (CTT)

First, CTT was used to evaluate the scales and item characteristics using SPSS 20.0 (IBM, Chicago, IL, USA). Item means were calculated to assess item difficulty. Cronbach’s alpha coefficient (α) was computed to assess scale internal consistency; values greater than 0.70 are deemed acceptable for general research purposes [34]. Item discrimination was evaluated using corrected item total correlations (CITC) that were calculated by the correlation coefficients between the scores on the item and the sum of scores of all the other items in a scale. Poorly discriminating items were identified with CITC lower than 0.30 [35]. The intraclass correlation coefficient with a two-way random model was computed to determine test-retest reliability; a minimum threshold of 0.70 was considered adequate [36].

#### Item response modeling (IRM)

Exploratory factor analysis was used to examine the primary assumption of IRM, unidimensionaltiy, for each subscale. The assumption of unidimensionalty was met if the scree plots showed one dominant factor, the first factor explained at least 20% of scale variance, and the factor loadings were >0.30 [37].

IRM models illustrate respondents’ latent trait based on their patterns of item responses. Both respondents’ trait levels and items’ psychometric properties are specified in IRM models. The degree of difficulty in agreeing with an item or endorsing a category is modeled as a function of person trait and item parameters. There are different mathematical forms of item characteristic functions and the number of parameters estimated for IRM models, but all IRM models include one or more item parameters to describe the probability of a certain score on an item, given a person’s latent traits [38, 39].

Polytomous IRM models, are used when items present multiple response choices, such as in attitude surveys and personality assessment tests [40, 41]. Only polytomous models are discussed here because the self-efficacy scale items present three response categories. Polytomous models model the probability for any item of endorsing one response category over another. Polytomous models include additional parameters, referred to as *category boundary*, *threshold parameter* or *step difficulty* which indicate the probabilities of responding at or above a given category. For an item with *k* response options, there are *k*–1 thresholds between the response options. For example, an item with three response options (*I am not sure*, *I am a little bit sure*, and *I am very sure*) will require two threshold estimates: (1) the step from “*I am not sure*” to “*I am a little bit sure*”, and (2) from “*I am a little bit sure*” to “*I am very sure*”, One goal of fitting a polytomous model is to determine the location of such thresholds along the latent trait continuum.

Due to the number of the subscales and responses, multidimensional polytomous models, was selected to assess respondents’ latent traits. Two polytomous models were considered: the partial credit (PCM) [42] and the rating scale models (RSM) [43, 44]. RSM is a special case of the PCM where the response scale is fixed for all items. That is, the response threshold parameters are assumed to be identical across items. For the present study, the final choice of a model was determined by comparing the deviance of the two competing multidimensional polytomous models using a Chi-square test.

Item fit was evaluated using infit and outfit mean square item fit indices (MNSQ) which have non-negative values. Infit is an information-weighted form of outfit. Infit MNSQ (information-weighted fit statistic) and outfit MNSQ (outlier-sensitive fit statistic) are based on information-weighted sum of squared standardized residuals and non-weighted sum of squared standardized residuals, respectively [45]. An infit or outfit MNSQ value of around one suggests the observed variance is similar to the expected variance. Mean square values greater than one or smaller than one indicate the observed variance is greater or smaller than expected, respectively. Infit or outfit MNSQ values greater than 1.3 indicate poor item fit when sample size is smaller than 500 [46]. With respect to thresholds, outfit MNSQ values greater than 2.0 indicate misfits, identifying candidates for collapsing with a neighboring category [45, 47].

Item-person maps, often called Wright maps (with units referred to as *log odds*), present both the distributions of scale items with that of the respondents on the same scale. Person, item and threshold estimates were placed in the same map where “*x*” on the left side represented the distribution of person trait estimates along the self-efficacy continuum with the student scoring the highest self-efficacy placed at the top of the figure. Item and threshold difficulties were presented on the right side, with the more difficult response items and categories placed at the top. I_*k*_ denotes threshold *k* for item I.

#### Differential item functioning (DIF)

Participants with the same underlying trait level may have different probabilities of endorsing an item. DIF is an indicator when an item performed differently between groups of individuals. For example, a finding of DIF by sex means that a male and a female with the same latent trait level responded differently to an item, indicating that the respondents’ interpretation of the item differed for men and women.

DIF was assessed by adding a group main effect and an item-by-group interaction term to the model [27, 48–50]. Whether an overall scale demonstrated DIF was indicated by a significant chi-square for the item-by-group interaction term. The ratio of the item-by-group parameter estimates to the corresponding standard error identified which items displayed DIF. DIF was indicated when the estimate to standard error ratio exceeded 1.96. The magnitude of DIF was determined by examining the differences of the item-by-group interaction parameter estimates. Because the sum of the parameters was constrained to be zero, if only two groups were considered, the magnitude of DIF difference was twice the estimates of the first reference group. For example, the estimate of the sex by item effect for Item 1 for males was −0.2, and then the estimate of the group by item effect for Item 1 for females was 0.2. The difference in item difficulty between older and younger children was −0.4. If comparison was made among three or more groups, the magnitude of DIF was the differences in estimates of the corresponding groups. Items that displayed statistically significant DIF were placed into one of three categories depending on the effect size: small DIF (difference < 0.426), intermediate DIF (0.426 < difference < 0.638), and large DIF (difference > 0.638) [51, 52]. ACER ConQuest [53] was used for all IRM analyses.

## Results

### Descriptive statistics

Participants’ characteristics are shown in Table 1. Thirty-five children (4.4%) did not complete any of the items and were excluded from analyses, resulting in a sample of 763 children with 55.2% boys. Participants were classified into younger children aged 8-10 years (43.5%) and older children aged 11-13 years (56.5%). Body weight status was categorized into three groups with 96 (13.1%) underweight children, 417 (56.8%) children with healthy weight, and 221 (30.1%) overweight/obese children.

**Table 1:** Participants’ characteristics (*N* = 763)

Demographic characteristics	n	%
Sex		
Boys	421	55.2
Girls	342	44.8
Age (yrs)		
8-10	332	43.5
11-13	431	56.5
Body status		
Underweight	96	13.1
Healthy weight	417	56.8
Overweigh/obesity	221	30.1

### Classical test theory (CTT)

The percentages of variance explained by the one-factor solution were 39.7%, 49.0%, 54.5% and 49.7% for FSE, VSE, WSE and PASE, respectively. Each scree plot revealed one dominant factor and factor loadings were higher than 0.30 for all the scales.

As presented in Table 2, CTT revealed that item difficulty (item means) ranged from 1.51 (0.76) to 2.59 (0.65) based on the scale ranging from 1 to 3, indicating that on average the responses were moderately difficult to agree with. Internal consistencies were excellent for PASE (α = 0.91), good for FSE (α = 0.86) and VSE (α = 0.85), and adequate for WSE (α = 0.79). CITCs were acceptable to high (0.40 to 0.74). The test-retest reliabilities were acceptable: 0.80 for FSE, 0.78 for VSE, 0.71 for WSE, and 0.79 for PASE.

**Table 2:** Item description, and estimated of differential item functioning where significant

Item	Item Question	CTT		IRM				
		Mean (SD)	CITC	Difficulty differences				
				Boy - Girl^a^	8-10 yo – 11-13 yo^b^	UW - HW^c^	UW - OW/OB^d^	HW - OW/OB^e^
Fruits self-efficacy (Cronbach’s alpha = 0.86)								
12	… eat 1 portion of fruit most times when you eat at a fast food place.	1.51 (0.76)	0.49					
11	… eat 1 portion of fruit at a fast food place at least one time.	1.55 (0.78)	0.49	−0.47**	−0.30*		0.20*	0.21*
10	… eat 1 portion of fruit for dinner at home at least one time.	1.84 (0.89)	0.48	−0.17*				0.27*
3	…ask someone in your family to buy 3 fruit at least every week.	2.02 (0.85)	0.64					
4	… eat 1 portion of fruit at lunch at least one time on a school day.	2.06 (0.87)	0.43					
6	… eat 1 portion of fruit for lunch most non-school days, including weekends.	2.09 (0.85)	0.59					
2	…ask someone in your family to buy 3 fruit at least one time.	2.13 (0.83)	0.59			0.18*		−0.19*
1	…ask someone in your family to buy your favorite fruit every week.	2.14 (0.78)	0.54	0.19*				
5	… eat 1 portion of fruit for lunch at least one time on a non-school day, including weekends.	2.23 (0.84)	0.59	0.19*	0.18*			
8	…eat 1 portion of fruit for a snack at home at least 4 days a week.	2.24 (0.83)	0.55	0.23*				
9	…ask someone in your family to serve 1 fruit instead of your usual dessert for dinner most nights.	2.26 (0.83)	0.52			−0.16*		
7	… eat 1 portion of fruit for a snack at home at least one time.	2.35 (0.79)	0.55	0.36*	0.25*		−0.32*	−0.25*
Vegetables self-efficacy (Cronbach’s alpha = 0.85)								
6	… eat 3 portions of vegetables at least 4 days a week, even when you are stressed.	1.80 (0.83)	0.61	−0.20*	−0.20*	0.48**	0.45**	
7	… eat 1 portion of a vegetable most times when you eat at a fast food place.	1.91 (0.84)	0.56					
2	…cut up 1 portion of a vegetable and eat it with a dip for a snack at least one time.	1.91 (0.85)	0.59			0.20*	0.21*	
4	… ask someone in your family to serve 2 vegetables for dinner most nights.	1.94 (0.84)	0.67			−0.17*	−0.28*	
3	… ask someone in your family to serve 2 vegetables for dinner at least one time.	2.03 (0.85)	0.68					
8	… eat 1 portion of a vegetable most times when you eat at a cafeteria type restaurant.	2.07 (0.85)	0.55					
5	…eat 3 portions of vegetables at least 4 days a week.	2.18 (0.84)	0.59		0.19*		0.27*	0.21*
1	…eat 1 portion of a vegetable at lunch at least one time on a school day.	2.37 (0.80)	0.45			−0.46**	−0.44**	
Water self-efficacy (Cronbach’s alpha = 0. 79)								
5	... drink 6 glasses or bottles of water for at least one day.	2.21 (0.85)	0.55			0.47**	0.44**	
4	…drink 6 glasses or bottles of water for at least 4 days a week.	2.26 (0.80)	0.65			0.19*		
3	… drink only water whenever you are thirsty for at least 4 days a week.	2.26 (0.82)	0.55		−0.29*			
1	…drink only water whenever you are thirsty for at least one day.	2.42 (0.77)	0.43	0.25*		−0.39*	−0.38*	
2	… drink 4 glasses or bottles of water for at least one day.	2.48 (0.75)	0.65		0.32*			
Physical Activity self-efficacy (Cronbach’s alpha = 0.91)								
11	… be physically active more than 30 min for at least 4 days a week, even when the weather outside is bad.	1.83 (0.82)	0.70					
6	… ask your friends to be physically active with you more than 30 min for at least 4 days a week.	1.87 (0.85)	0.69			−0.21*		
10	… be physically active more than 30 min for at least 4 days a week when your friends want to do something else.	1.90 (0.84)	0.73	−0.22*	−0.25*			
8	… be physically active more than 30 min for one day, even when you have lots of other things to do.	1.93 (0.84)	0.74				−0.26*	
7	… be physically active more than 30 min for one day, even when you have homework.	1.94 (0.85)	0.71					
9	… be physically active more than 30 min for at least 4 days a week, even when you have lots of other things to do.	1.98 (0.85)	0.74		−0.25*			
4	… the ability to play team sports like basketball, soccer or softball really well.	2.05 (0.84)	0.60	−0.71***			0.28*	0.38*
5	… ask your friends to be physically active with you more than 30 min for at least one day.	2.20 (0.83)	0.56		0.19*		0.26*	0.21*
12	… be physically active more than 30 min for most nonschool days, including weekends.	2.26 (0.80)	0.58					
2	… the ability to do physical activities like running, dancing, bicycling, or jumping rope really well.	2.24 (0.75)	0.58	0.44**	−0.22*		−0.42*	−0.50**
3	… the ability to play team sports like basketball, soccer or softball.	2.29 (0.81)	0.54	−0.79***	0.27*	0.31*	0.54**	0.23*
1	… the ability to do physical activities like running, dancing, bicycling, or jumping rope	2.59 (0.65)	0.40	0.79***	0.23*		−0.43*	−0.46**

Note: *CTT* classical test theory, *IRM* item response model, *CITT* corrected item total correlations, *SD* standard deviation, *UW* underweight, *HW* healthy weight, *OW* overweight, *OB* obesity

IRM difficulty difference was assessed to indicate whether an item performed differently between groups of individuals, including sex, age, and body weight status groups. Specifically

^a^ Positive numbers, easier for girls; Negative numbers, easier for boys

^b^ Positive numbers, easier for 11-13-year-old children; Negative numbers, easier for 8-10-year-old children

^c^ Positive numbers, easier for children with healthy weight; Negative numbers, easier for underweight children

^d^ Positive numbers, easier for overweight/obese children; Negative numbers, easier for underweight children

^e^ Positive numbers, easier for overweight/obese children; Negative numbers, easier for children with Healthy weight

*small effect (difference < 0.426)

**moderate effect (0.426 < difference < 0.638)

***large effect (difference > 0.638)

### IRM model fit

The relative fit of multidimensional RSM and multidimensional PCM was evaluated by considering the deviance difference, where *df* was equal to the difference in the number of estimated parameters between the two models. The chi-square (χ^2^) deviance statistic was calculated by considering differences in model deviances (RSM: 46,107.92; PCM: 45,903.92) and differences in numbers of parameters (RSM: 48; PCM: 84) for the nested models. The chi-square test of the deviance differences showed that RSM significantly reduced model fit (∆ deviance = 204.01, *df* = 36, *p* < 0.0001). Thus, the analyses indicated that the multidimensional RSM did not perform as well as the multidimensional PCM. As a result, further analyses reflect those from PCM.

### Item fit

A summary of misfit indicators (MNSQ) and item difficulties are shown in Table 3. The MNSQ values greater than 1.3 indicate poor item fit. One VSE item (item 1, infit mean square = 1.60) and one PASE item (infit mean square for item 1 = 1.33) did not meet the recommended criterion value of 1.3. Both items were also misfits in the differential item functioning analyses when the subgroups were students’ sex (VSE Item 1 infit mean square = 1.35; PASE Item 1 infit mean square = 1.32), age (VSE Item 1 infit mean square = 1.63; PASE Item 1 infit mean square = 1.68), and weight status (VSE Item 1 infit mean square = 1.39; PASE Item 1 infit mean square = 1.46).

**Table 3:** Item description, item difficulty, and misfit item(s)

Item	Item questions	All	Boys	8-10 yrs	Underweight	Healthy Weight	Overweight /Obesity
Fruits self-efficacy (Cronbach’s alpha = 0.86)							
12	…eat 1 portion of fruit most times when you eat at a fast food place.	1.171	−0.197	−0.159	0.031	0.076	−0.107
11	…eat 1 portion of fruit at a fast food place at least one time.	1.052	−0.233	−0.149	0.063	0.073	−0.136
10	…eat 1 portion of fruit for dinner at home at least one time.	0.402	−0.085	0.075	−0.005	0.138	−0.133
3	…ask someone in your family to buy 3 fruit at least every week.	0.011	−0.001	−0.030	0.065	−0.061	−0.004
4	…eat 1 portion of fruit at lunch at least one time on a school day.	−0.049	−0.019	−0.075	0.085	−0.042	−0.043
6	…eat 1 portion of fruit for lunch most non-school days, including weekends.	−0.100	0.061	−0.018	0.009	0.003	−0.011
2	…ask someone in your family to buy 3 fruit at least one time.	−0.189	−0.051	0.029	0.053	−0.123	0.070
1	…ask someone in your family to buy your favorite fruit every week.	−0.256	0.096	0.078	−0.018	0.042	−0.023
5	…eat 1 portion of fruit for lunch at least one time on a non-school day, including weekends.	−0.400	0.096	0.090	−0.017	−0.057	0.074
8	…eat 1 portion of fruit for a snack at home at least 4 days a week.	−0.436	0.115	0.061	−0.017	−0.032	0.050
9	…ask someone in your family to serve 1 fruit instead of your usual dessert for dinner most nights.	−0.487	0.039	−0.025	−0.115	0.041	0.074
7	…eat 1 portion of fruit for a snack at home at least one time.	−0.719	0.179	0.123	−0.133	−0.057	0.190
Vegetables self-efficacy (Cronbach’s alpha = 0.85)							
6	…eat 3 portions of vegetables at least 4 days a week, even when you are stressed.	0.556	−0.098	−0.102	0.308	−0.171	−0.137
7	…eat 1 portion of a vegetable most times when you eat at a fast food place.	0.267	0.017	0.012	−0.049	−0.044	0.092
2	…cut up 1 portion of a vegetable and eat it with a dip for a snack at least one time.	0.250	−0.074	−0.029	0.136	−0.062	−0.074
4	…ask someone in your family to serve 2 vegetables for dinner most nights.	0.214	0.016	0.027	−0.151	0.021	0.130
3	…ask someone in your family to serve 2 vegetables for dinner at least one time.	0.028	0.000	0.020	−0.030	0.041	−0.011
8	…eat 1 portion of a vegetable most times when you eat at a cafeteria type restaurant.	−0.110	0.095	−0.016	−0.022	0.007	0.015
5	…eat 3 portions of vegetables at least 4 days a week.	−0.363	−0.014	0.095	0.108	0.050	−0.158
1	…eat 1 portion of a vegetable at lunch at least one time on a school day.	−0.841^a^	0.057^c^	−0.007^e^	−0.300^g^	0.157^g^	0.143^g^
Water self-efficacy (Cronbach’s alpha = 0. 79)							
5	...drink 6 glasses or bottles of water for at least one day.	0.345	−0.042	−0.049	0.303	−0.164	−0.139
4	…drink 6 glasses or bottles of water for at least 4 days a week.	0.168	−0.068	0.032	0.103	−0.087	−0.016
3	…drink only water whenever you are thirsty for at least 4 days a week.	0.167	−0.032	−0.145	−0.082	0.045	0.036
1	…drink only water whenever you are thirsty for at least one day.	−0.268	0.123	0.001	−0.258	0.135	0.124
2	…drink 4 glasses or bottles of water for at least one day.	−0.413	0.019	0.162	−0.066	0.071	−0.005
Physical Activity self-efficacy (Cronbach’s alpha = 0.91)							
11	…be physically active more than 30 min for at least 4 days a week, even when the weather outside is bad.	0.748	0.035	−0.077	0.097	−0.052	−0.044
6	…ask your friends to be physically active with you more than 30 min for at least 4 days a week.	0.623	−0.067	0.058	−0.106	0.106	0.001
10	…be physically active more than 30 min for at least 4 days a week when your friends want to do something else.	0.547	−0.109	−0.124	0.028	0.009	−0.038
8	…be physically active more than 30 min for one day, even when you have lots of other things to do.	0.462	−0.006	−0.075	−0.135	0.012	0.122
7	…be physically active more than 30 min for one day, even when you have homework.	0.397	−0.019	−0.058	−0.068	0.011	0.058
9	…be physically active more than 30 min for at least 4 days a week, even when you have lots of other things to do.	0.331	0.055	−0.125	−0.024	0.052	−0.027
4	…the ability to play team sports like basketball, soccer or softball really well.	0.104	−0.357	0.014	0.057	0.163	−0.220
5	…ask your friends to be physically active with you more than 30 min for at least one day.	−0.277	0.068	0.094	0.107	0.050	−0.157
12	…be physically active more than 30 min for most nonschool days, including weekends.	−0.450	0.180	0.152	0.001	0.036	−0.038
2	…the ability to do physical activities like running, dancing, bicycling, or jumping rope really well.	−0.453	0.219	−0.108	−0.114	−0.191	0.304
3	…the ability to play team sports like basketball, soccer or softball.	−0.516	−0.395	0.133	0.286	−0.028	−0.258
1	…the ability to do physical activities like running, dancing, bicycling, or jumping rope	−1.515^b^	0.396^d^	0.117^f^	−0.129^h^	−0.167^h^	0.296^h^

Item fit was evaluated using infit and outfit mean square item fit indices (MNSQ), which are based on information-weighted sum of squared standardized residuals and non-weighted sum of squared standardized residuals, respectively. Infit or outfit MNSQ values greater than 1.3 indicate poor item fit

^a^ Misfit item (item 1) outfit mean square = 1.60

^b^ Misfit item (item 1) outfit mean square = 1.33

^c^ Misfit item (item 1) outfit mean square = 1.35

^d^ Misfit item (item 1) outfit mean square = 1.32

^e^ Misfit item (item 1) outfit mean square = 1.63

^f^ Misfit item (item 1) outfit mean square = 1.68

^g^ Misfit item (item 1) outfit mean square = 1.39

^h^ Misfit item (item 1) outfit mean square = 1.46

### Item-person fit Wright map

Table 4 presents the PCM item-person maps. The participants’ self-efficacy estimates (confidence for fruit, vegetable, water intakes, and PA engagement), and the item and item threshold difficulty distributions are on the same logit scale. The difficulty distribution is ideally presented with a normal distribution from −3.0 to +3.0. As shown in the figure, FSE and VSE approached a normal distribution. There were small portions of participants with higher and lower levels of WSE and PASE (logits >3.0/ < −3.0).

**Table 4:** Wright map of item thresholds for FSE, VSE, WSE, and PASE

Logit	Latent Ability Distribution				Item Distribution				Item Threshold Distribution			
	Fruit	Vegetable	Water	Physical activity	Fruit	Vegetable	Water	Physical activity	Fruit	Vegetable	Water	Physical activity
	Extremely high self-efficacy								Extremely difficult to endorse response			
5												
4				X								
			X	X								
			X	X								
			X	XX								
			XX	X								2.2
3		X	XX	X								
		X	XX	X								
	X	XX	XXXX	X					1.2			1.2 12.2
	XX	X	XXX	XX							4.2	4.2 11.2
2	X	XX	XXXX	XX								10.2
	XX	XXX	XXXX	XX					10.1			5.2 7.2 8.2
	XXX	XXX	XXXX	XXX						4.2	3.2	3.2 6.2 9.2
	XXX	XX	XXXX	XXX						6.2		
	XXXX	XXXX	XXXX	XXX	11 12				11.1 12.1	3.2 5.2		
1	XXXXX	XXXXX	XXXXX	XXXX					2.2	2.2 7.2 8.2	1.2	
	XXXXXX	XXXXX	XXXXXX	XXXXX				6 11	3.2 4.1	1.2	2.2 5.2	
	XXXXXXX	XXXXX	XXXXXX	XXXX	10	6		7 8 10	7.2			
	XXXXXXX	XXXXXX	XXXXXX	XXXXXX		2 4 7	3 4 5	9	5.1 6.2 8.2 9.2			
0	XXXXXXXXXX	XXXXXXXX	XXXXX	XXXXXX	3 4	3		4				
	XXXXXXXX	XXXXXXXX	XXXXX	XXXXXXX	1 2 6	8	1	5	5.2 6.1 8.1 9.1			
	XXXXXX	XXXXX	XXXX	XXXX	5 8 9	5	2	2 3 12	7.1			
	XXXXXX	XXXXX	XXX	XXXXX	7				3.1 4.2	1.1	2.1 5.1	
	XXXXX	XXXXX	XXX	XXXX		1			2.1	2.1 7.1 8.1	1.1	
-1	XXX	XXXX	XX	XXXX					11.2 12.2	3.1 5.1		
	XXX	XXX	X	XXX						6.1		
	XX	XX	X	XX				1		4.1	3.1	3.1 6.1 9.1
	XX	XX	X	X					10.2			5.1 7.1 8.1
-2	X	X		X								10.1
	X	X	X	X							4.1	4.1 11.1
		X							1.1			1.1 12.1
		X		X								
-3		X		X								
												2.1
				X								
-4												
	Extremely low self-efficacy								Extremely easy to endorse response			

On the left side of figure, the participants’ self-efficacy estimates were placed on the map with “X” following the outermost left column of logit scale.

“X” indicated the trait estimates of persons. Each ‘X’ represents 8.1 cases. Higher self-efficacies were placed at the top of the column, while, the lower self-efficacies were located at the bottom of the column

Item and threshold difficulties were presented on the right side of figure, with the top locations indicating the more difficult to endorse responses

The items were distributed in the centre of the Wright diagram. Item difficulties showed that the logits ranged from – 0.719 to 1.171 for FSE, from −0.841 to 0.556 for VSE, from −0.413 to 0.345 for WSE, and from −1.515 to 0.748 for PASE, respectively. The distributions nearly overlapped between item threshold and person measures (indicating the full distribution of individuals was measured by items across the whole distribution, as desired) for three of the self-efficacy scales, except VSE. Participants at the lower and higher ends of VSE did not coincide with the item’s first and second threshold.

### Differential item functioning (DIF)

#### Children’s sex groups

Item difficulty differences across sex, age, and body weight status groups are presented in Table 2. Small DIF was detected for items 1, 5, 7, 8, 10 as well as moderate DIF for item11 in FSE across sex groups. Among these items, boys found it easier to endorse items 10 and 11, but more difficult to endorse the others. Only item 6 in VSE had significant DIF by sex at −0.20, a small DIF effect: it was easier for boys to endorse item 6. Item 1 of WSE was detected with a small DIF effect, easier for girls. Five items had significant DIF (small: item 10; moderate: item 2; large: items 1, 3, and 4) in PASE. It was easier for boys to endorse items 3, 4, and 10.

#### Children’s age groups

Older children aged 11-13 years were more likely to endorse item 5 (small DIF at 0.18) and item 7 in FSE (small DIF at 0.25), but less likely to endorse item 11 with small DIF at −0.30. Two items had small DIF in VSE (items 5 and 6) and WSE (items 2 and 3) among different age groups, respectively. Older children found that somewhat easier to endorse item 5 of VSE and item 2 of WSE. Small DIF was indicated for six items (items 1, 2, 3, 5, 9, 10) of PASE between younger and older children. It was easier for older children to endorse items 1, 3, and 5.

#### Children’s body weight status

Between underweight and healthy weight children, small DIF was detected for items 2 (easier for healthy weight children) and 9 of FSE, item 2 (easier for healthy weight children) and 4 of VSE, items 1 and 4 (easier for healthy weight children) of WSE, and items 3 (easier for healthy weight children) and 6 of PASE as well as medium DIF detected for items 1 and 6 (easier for healthy weight children) of VSE, item 5 (easier for healthy weight children) of WSE. In comparison of underweight and overweight/obese children, items 7 (easier for underweight children) and 11 of FSE, items 2, 4 (easier for underweight children) and 5 of VSE, item 1 (easier for underweight children) of WSE, and items1, 2, 4, 5 and 8 of PASE (easier for underweight children for item 1, 2, and 8) were examined with small DIF; items 1 (easier for underweight children) and 6 of VSE, item 5 of WSE, and item 3 of PASE showed medium DIF. Between healthy and overweight and obese children, small DIF was indicated for items 2, 7, 10, and 11 of FSE (easier for healthy children for item 2 and 7), items 5 of VSE, and items 3, 4, 5 of PASE; and medium DIF were indicated for items 1 and 2 (both easier for healthy children) of PASE. No large DIF was found across different body weight status groups.

## Discussion

The present study investigated the psychometric properties of FSE, VSE, WSE and PASE scales using CTT and IRM, and their stability across sex, age and body weight status groups based on IRM using the partial credit model. CTT results showed that the examined scales had adequate to excellent internal consistency and adequate test-retest reliability. The item difficulties were moderately easy to difficult. Items in the scales were considered discriminating. The symmetric distribution of items and item thresholds for individuals from the Wright map indicated the utilization of three-point responses nearly covered the participants from low to high levels of each self-efficacy scale except VSE, suggesting the items in VSE should be revised or new ones developed to cover the more difficult and easy levels.

One item (item1) in VSE and one items (item1) in PASE were identified as misfit items. These items also exhibited DIF across different groups. Item 1 of VSE (i.e., “How sure are you that you can eat 1 portion of a vegetable at lunch at least one time on a school day?”) and item 1 of PASE (i.e., “How sure are you that you have the ability to do physical activities like running, dancing, bicycling, or jumping rope?”) showed moderate DIF on the basis of children’s body weight status. Compared with overweight/obese children, underweight children tended to have 1 portion of a vegetable at least once on a school day. Children with healthy weight were more likely to engage in various kinds of PA than overweight and obese children. These findings suggest children’s perceived confidence to comply with the healthy lifestyle differed across different body weight status, consistent with the previous studies [25, 54, 55]. Since these two items did not behave the same way across these groups, they should be substantially revised or deleted from the scales.

DIF presented distinct difficulties by children’s sex groups. Given items with small DIF are generally not of major concern [56], we only discuss items with medium/large DIF because they require more attention in the future studies. Ignoring small DIF effects, there was moderate DIF for item 11 of FSE, and item 2 of PASE, and large DIF for items 3 and 5 of PASE. Boys showed higher confidence that they could participate in team sports (e.g., basketball, softball) than girls, but not in flexibility/rhythm-related activities (e.g., dancing, jumping rope). These DIF suggest sex-specific tailoring of an intervention to boys and girls based on their differences of food and activity preferences, as suggested by existing research [57, 58].

DIF across demographic variables could be due to differences in ability to comprehend the meaning of the specific items or actual differences in the efficacy level to adopt healthy eating behaviors or engage in PA. Moderate DIF across body weight status groups and moderate to large DIF across sex groups indicate the need to re-check and revise items to produce non-significant DIF or reduce DIF to a considerably lower level [59]. Developing the sex and body weight status specific self-efficacy scales should be considered.

VSE items and thresholds did not cover the higher and lower difficult to endorse ends of confidence. This may require rewriting existing items or adding new items to extend the end of the distribution of items and thresholds. For example, a VSE item at average difficulty, “I can eat 1 portion of a vegetable at lunch at least one time on a school day”, might be revised into “I can eat 1 portion of a vegetable at lunch at least three times on school days” , which would appear to have greater difficulty. An item with large difficulty, e.g., “I can eat 3 portions of vegetables at least 4 days a week”, could be transformed to possibly low difficulty, e.g., “I can eat 3 portions of vegetables at least one day a week”.

In the study, WSE contained 5 items and the logits of item difficulties ranged from −0.413 to 0.345. WSE showed narrower item distribution compared with the other three ones. To cover a wider range of latent trait, more diverse WSE items should be developed in future studies. For example, items addressing confidence in overcoming different types of barriers to have more water [32] (e.g., social impediments [60] referred to as coping SE [61], or emotional state). Additionally, types of item which could enhance the distributional properties could also be examined in the future.

Several limitations of the study should be mentioned. Even though existing and previously validated instruments were used and demonstrated good internal consistency in this study, validity of the scales are not available among the target children. Further validation studies should be implemented to evaluate the application of scales in different cultural settings among Chinse children (e.g., children from urban and rural areas in mainland China). Furthermore, IRM’s complexity requires a large sample size. Recommendations have been ranged from 200 per group [62] to 500 per group [63]. Possible limitations of small sample size should be acknowledged in the current study. Further investigation should retest the findings by recruiting more participants. Moreover, further investigation could be undertaken with other DIF-detection procedures (e.g., non-uniform differential item functioning).

## Conclusion

FSE, VSE, WSE and PASE demonstrated acceptable factorial validity, test-retest reliability, and adequate to excellent internal consistency by CTT. IRM provides useful insights on item difficulty estimates that were not dependent on the sample. The latent variables indicated adequate fit to the data, however, the items and thresholds did not adequately cover the easier and more difficult to endorse ends of VSE. A revised VSE questionnaire is needed to provide full range of self-efficacy difficulty estimates. Several items of the four examined self-efficacy scales exhibited moderate or large differential item functioning on the basis of children’s sex and body weight status. Additional psychometric work remains to be done while scales can be used in diverse groups with due caution. Further formative work for questionnaire is necessary.

## Abbreviations

BMI: Body mass index
CITC: Corrected item total correlations
CTT: Classical test theory
DIF: Differential item functioning
FSE: Self-efficacy for fruit
IRM: Item response modeling
MNSQ: Mean square item fit indices
PA: Physical activity
PASE: Self-efficacy for physical activity
PCM: Partial credit model
RSM: Rating scale model
SES: Socio-economic status
TRT: Test-retest reliability
VSE: Self-efficacy for vegetable
WSE: Self-efficacy for water
